# Intraosseous Bone Marrow Concentrate for Hip Osteoarthritis: A Case Series

**DOI:** 10.7759/cureus.97885

**Published:** 2025-11-26

**Authors:** Rebecca L Cox, Jasper Tseng, Chris Williams

**Affiliations:** 1 Physical Medicine and Rehabilitation, MedStar National Rehabilitation Hospital, Washington, DC, USA; 2 Physical Medicine and Rehabilitation, Emory University School of Medicine, Atlanta, USA; 3 Physical Medicine and Rehabilitation, Interventional Orthopedics of Atlanta, Atlanta, USA

**Keywords:** bmc, bone marrow concentrate, hip oa, hip osteoarthritis, intra-articular injection, intraosseous injection, orthobiologic

## Abstract

Hip osteoarthritis (OA) is a prevalent cause of pain and disability in the US, with continued progression often necessitating total hip replacement surgery. However, postoperative complications and persistent pain can occur, compelling the need for alternative long-term management strategies. Advances in orthobiologics, such as bone marrow concentrate (BMC) injections, offer a non-surgical alternative for hip OA treatment that can facilitate pain management and disease modification. Recent case studies show significant improvement in pain, function, and quality of life following intra-articular BMC injections, but there is limited literature on the effect of intraosseous (IO) orthobiologic injections for hip OA. We hope to provide insight into this topic of IO BMC injections under fluoroscopic guidance for hip OA with our case series that followed four males and one female with an average age of 67.6 years and a Tonnis score of 2. Primary outcomes included change in the visual analog scale (VAS) pain score and subjective functional improvement using the single-assessment numeric evaluation score. Secondary outcomes studied included adverse events and safety. Despite varying follow-up durations, all patients experienced greater than 50% improvement in function, with the average pain reduction on VAS from 7.6 to 2.4. No adverse effects were reported. IO BMC injections appear safe and effective for hip OA. Further investigation is warranted, including randomized controlled trials with larger samples and consistent follow-ups to validate these findings and assess long-term efficacy through follow-up imaging.

## Introduction

Hip osteoarthritis (OA) is a common source of pain and dysfunction in the adult population [1]. The age-standardized incidence rate (ASIR) of hip OA has increased worldwide from 1990 to 2019, with the rate adjusted for age climbing from about 17 to nearly 19 cases for every 100,000 people [2]. Risk factors for hip OA include older age, female sex, obesity, diet, prolonged lifting and standing, trauma, and genetic factors [1,3,4].

Current treatment options for hip OA are limited, with end-stage disease often necessitating total hip arthroplasty (THA) [5]. Even after THA, surgical complications and continued pain after recovery can still occur. Moreover, the healthcare cost and burden associated with THA are growing. In less than a decade, between 1997 and 2004, the annual total cost of joint replacements almost tripled, rising from $7.9 billion to $22.6 billion [6].

Current routine non-surgical treatment options for hip OA include nonsteroidal anti-inflammatory drugs (NSAIDs), intra-articular (IA) injections, and physical therapy (PT). IA injections, including corticosteroids and hyaluronic acid, are frequently used for OA of the knee, but data are limited on their efficacy for the hip [7]. Additionally, recent evidence has suggested that, unlike other large joints (i.e., knees and shoulders), hips can have an incidence of up to 20% of rapid necrosis of the joint after IA corticosteroid injection [8]. PT, including the use of hip mobilizations, has been shown to enhance patient outcomes by addressing a limited range of motion (ROM), a common issue in hip OA [9].

Recent developments in orthobiologics, like bone marrow concentrate (BMC) injections, have the potential to provide a new non-surgical option in the management of hip OA [10-12]. BMC is an autologous biologic product derived from the patient’s own bone marrow that contains a heterogeneous mix of platelets, growth factors, white blood cells, and mesenchymal stem cells (MSCs) capable of modulating inflammation and disease modification [13,14]. The stem cells may contribute to the observed functional improvements by their capacity to differentiate into bone-forming osteoblasts, cartilage-producing chondrocytes, and other mature tissue types. Progenitor cells further aid tissue regeneration by promoting angiogenesis, releasing growth factors such as bone morphogenetic protein (BMP)-2 and BMP-6, and activating quiescent cells. Platelets serve as a rich source of signaling molecules that stimulate cellular proliferation and differentiation. Lymphocytes facilitate the migration and expansion of endothelial progenitor cells, while granulocytes enhance new blood vessel formation through the secretion of vascular endothelial growth factor (VEGF) and help regulate inflammatory responses [15]. While IA injections deliver biologic material to the joint space, intraosseous (IO) injections target the subchondral bone, a region increasingly recognized as central to OA pathophysiology due to bone marrow lesions, altered remodeling, and nociceptive signaling [1]. This distinction may explain emerging reports that IO delivery provides more durable hip pain relief and improved joint function compared with IA injections alone [2,10,16-20].

The use of IO BMC therapy may therefore offer a less invasive and potentially disease-modifying alternative to THA, particularly for patients with early- to mid-stage disease [10]. Several recent studies have demonstrated promising improvements in pain, mobility, and radiographic markers following BMC administration, though evidence remains limited to small case series and very few randomized clinical trials [5-7,17-20].

As research continues to investigate novel treatment modalities for hip OA, it is essential to critically evaluate the effectiveness and safety of these interventions. Expanding the understanding of how biologic preparations like BMC interact with subchondral bone and cartilage can inform the design of future regenerative therapies and optimize delivery techniques. Understanding the mechanisms of action and the impact of interventions like BMC injections on hip OA can pave the way for more targeted and personalized treatment options for patients suffering from this condition. This case series aims to help determine the effect of IO BMC injections on pain and functional improvement in the treatment of hip OA by discussing five cases of BMC use for hip OA.

## Case presentation

This retrospective case series reviewed seven cases of IO BMC injections for hip OA conducted at a single outpatient private practice clinic from 2018 until 2024. Patients included were those who: 1) presented with hip pain due to primary OA confirmed on imaging, such as X-ray or magnetic resonance imaging (MRI), 2) failed conservative measures like PT, oral NSAIDs, and steroid injections, and 3) completed an IO injection with BMC. Patients with a concurrent diagnosis of avascular necrosis were excluded. Two of the seven IO BMC cases were excluded from analysis after MRI confirmed avascular necrosis. The remaining five patients with hip OA underwent an autologous IO BMC injection after noting only minimal relief from PT, oral NSAIDs, and steroid injections. The severity of hip OA was assessed with X-ray imaging and graded on the Tönnis scale. The average Tönnis score was 2. Patients were followed from one month to two years post-IO BMC injection and assessed for improvement in pain and function using the visual analog scale (VAS) score and subjective percentage improvement in function using the single assessment numeric evaluation (SANE) score.

All procedures were performed by a single fellowship-trained provider with significant expertise in managing musculoskeletal disorders. A detailed description of the aspiration and concentration of the bone marrow has been previously described in the literature [13]. To create the BMC, an 11-gauge trocar needle was manually inserted into the posterior superior iliac spine (PSIS), and a 20-30 cc syringe was attached to the trocar for aspiration of a total of 90 cc of bone marrow aspirate under ultrasound guidance from six to 10 different aspiration sites on the PSIS. The collected aspirate was then processed using sterile technique in a centrifuge to generate BMC. Cell characterization data were recorded, including total nucleated cell (TNC) counts and cell viability. Reported ranges from the patient records included TNC counts of 3.8×10⁸ to 2.0×10⁹ and cell viability of 85-95%. Next, the BMC was injected into the femoral head for an IO injection with an 11-gauge trocar under direct visualization using fluoroscopic guidance. A smaller volume of BMC was also injected into the femoral-acetabular joint as an IA injection. For patients who received a capsular distention weeks before their BMC injection, the rationale was based on published literature linking capsular adhesions, inflammatory changes, increased pain, and decreased joint mobility to limitations in range of motion (ROM) in large joints [21-24]. Capsular dilation was completed with a 10-15 mL mixture of 0.5% ropivacaine, platelet-rich plasma (PRP), and platelet-poor plasma injected into the symptomatic arthritic joint. PRP utilized for these cases was obtained as previously described [13,25-27]. In brief, 60-70 mL of whole blood was used to prepare 5 mL of leukocyte-poor PRP.

Case 1

**Figure 1:**
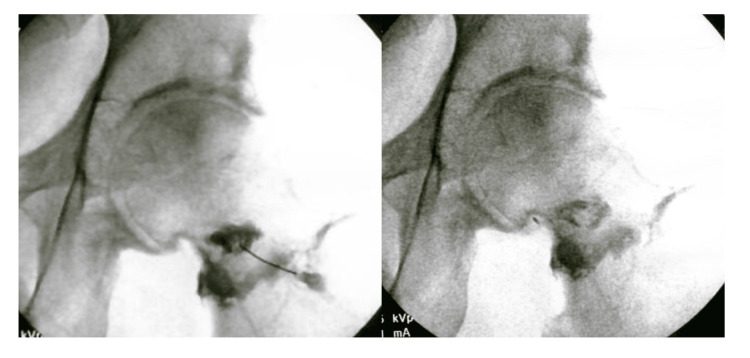
Patient 1 - left hip BMC injection Fluoroscopic images demonstrating the left intra-BMC concentrate injection in a 54-year-old male (Tönnis 1). Patient 1 reported VAS pain improved from 7 to 3 and a 60% SANE improvement. BMC, bone marrow concentrate; VAS, visual analog scale; SANE, single assessment numeric evaluation

Patient 1 was a 54-year-old male (Tönnis 1) with one year of left hip pain aggravated by walking and yardwork, associated with limited internal rotation (3-5°) and external rotation (40°). He hoped to return to racquetball and reported a baseline VAS of 7. MRI of the left hip showed bone marrow edema in the acetabulum, moderate-to-severe coaxially aligned mismatched CAM deformity of the femoral head and neck, subchondral pseudocystic changes, and mild OA. The patient underwent a capsular distention with 5 mL ropivacaine and 5 mL PRP, followed by aggressive ROM to address suspected capsular adhesions. Two weeks later, a BMC injection with TNC of 1.5×10⁹ and 87% viability was completed. The procedure involved sequential IO and IA injections under fluoroscopic guidance. The acetabular IO injection contained 5 cc BMC, 1 cc 10× super-concentrated PRP (SCP), 1 cc 10× platelet lysate (PLM), and 1 cc 7× PLM. The femoral head IO injection included 3 cc BMC, 1 cc 10× PLM, 1 cc 10× SCP, and 3 cc 7× PLM. The labral injection consisted of 1 cc BMC, 0.5 cc 10× SCP, and 0.5 cc 10× PLM. The IA injection used 1 cc BMC, 0.5 cc 10× SCP, and 0.5 cc 10× PLM, while the inferior capsule injection delivered 1.5 cc 7× PLM (Figure 1). At the two-month follow-up, patient 1 reported VAS improved from 7 to 3 and a 60% SANE improvement in function. After a fall two months later, a repeat MRI showed a larger cystic change in the acetabulum but less edema. The patient received an IA PRP injection two months after the fall with continued PT, which improved his symptoms at follow-up.

Case 2

**Figure 2:**
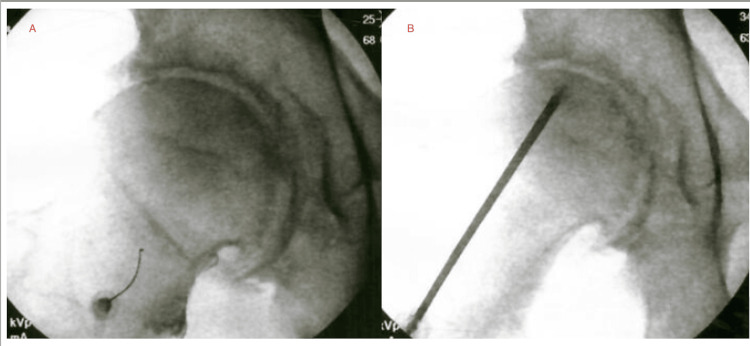
Patient 2 - right hip BMC injections Fluoroscopic-guided intra-articular (A) and intraosseous (B) BMC injections in a 65-year-old male (Tönnis 2). MRI revealed cartilage thinning and osteophyte formation. Patient 2 reported VAS improved from 7 to 2, and at least 50% SANE score improvement in function. BMC, bone marrow concentrate; VAS, visual analog scale; SANE, single assessment numeric evaluation; MRI, magnetic resonance imaging

Patient 2 was a 65-year-old male (Tönnis 2) with six months of worsening right hip pain, limited ROM, and nocturnal pain interfering with sleep. Physical exam showed external rotation (40°), internal rotation (15°), and pain with resisted hip flexion. MRI demonstrated mild femoroacetabular cartilage thinning, moderate marginal osteophyte formation, and small joint effusion consistent with mild-to-moderate OA. Baseline VAS was 7. A right hip IA injection was performed approximately two months after initial presentation. A capsular distention procedure was performed two months after the IA injection. One month after the capsular distention, BMC injections were performed under fluoroscopic guidance to the femoral head, acetabulum, labrum, IA space, and inferior capsule. The injectate consisted of high-density BMC (TNC count=2.0×10⁹; 85% viability) combined with 10 cc 7× PLM, 3 cc 10× PLM, and 3 cc 10× SCP (Figure 2). At the one-month follow-up, patient 2 reported VAS improved from 7 to 2 with at least 50% SANE functional improvement and resolution of nocturnal symptoms.

Case 3

**Figure 3:**
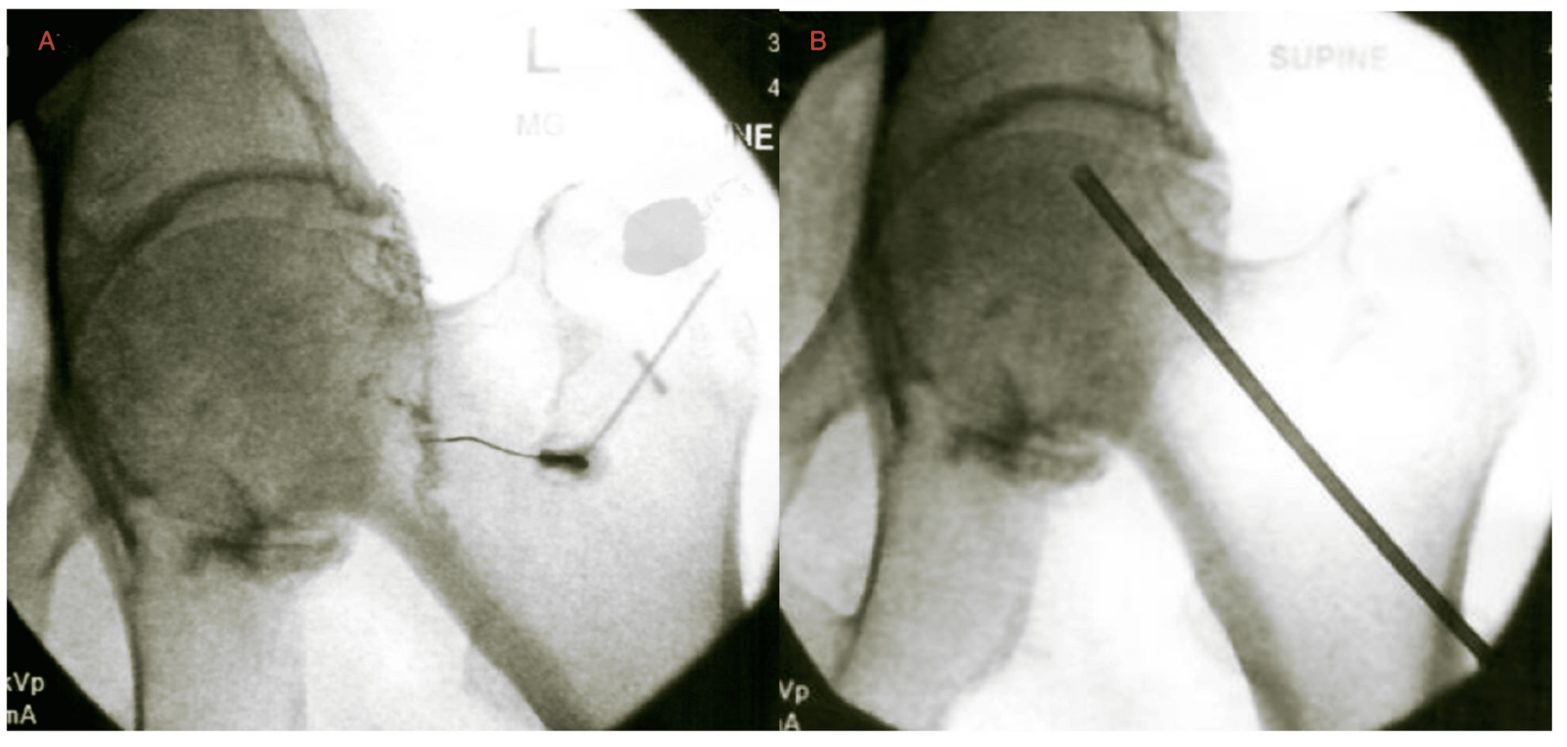
Patient 3 - left hip BMC injections Fluoroscopic-guided intra-articular (A) and femoral head intraosseous (B) BMC injection of a 68-year-old male (Tönnis 3) with subchondral cysts and labral degeneration. Post-treatment VAS improved from 7 to 1 with 60-70% SANE functional improvement. BMC, bone marrow concentrate; VAS, visual analog scale; SANE, single assessment numeric evaluation

Patient 3 was a 68-year-old male (Tönnis 3) with left hip pain, pelvic asymmetry, and limited external rotation (40°) and internal rotation (10°), accompanied by a positive Trendelenburg sign. MRI revealed severe degenerative joint disease with grade 4 chondromalacia, multiple subchondral cysts of the femoral head and acetabulum, and degenerative labral tearing. Baseline VAS was 7. A capsular distention was administered approximately one week after the initial presentation, followed by an IA PRP injection one week after the capsular distention, and BMC injections two weeks after the IA PRP. The BMC injection targeted the IA space, labrum, and femoral head (Figure 3). Treatment included an IA injection of 3 cc BMC, 2 cc 10× SCP, and 2 cc 10× PLM; a superior-posterior labral injection of 1 cc BMC; and a femoral head IO injection containing 3 cc BMC, 5 cc 7× SCP, 1 cc 10× SCP, and 1 cc 10× PLM. At the one-month follow-up, VAS improved to 1 with 60-70% SANE improvement in function and increased tolerance for activities like yardwork and prolonged standing. The patient still reported pain with sit-to-stand and entering his vehicle. Continued PT was recommended, and an additional IA PRP injection was completed six months after the BMC injections.

Case 4

**Figure 4:**
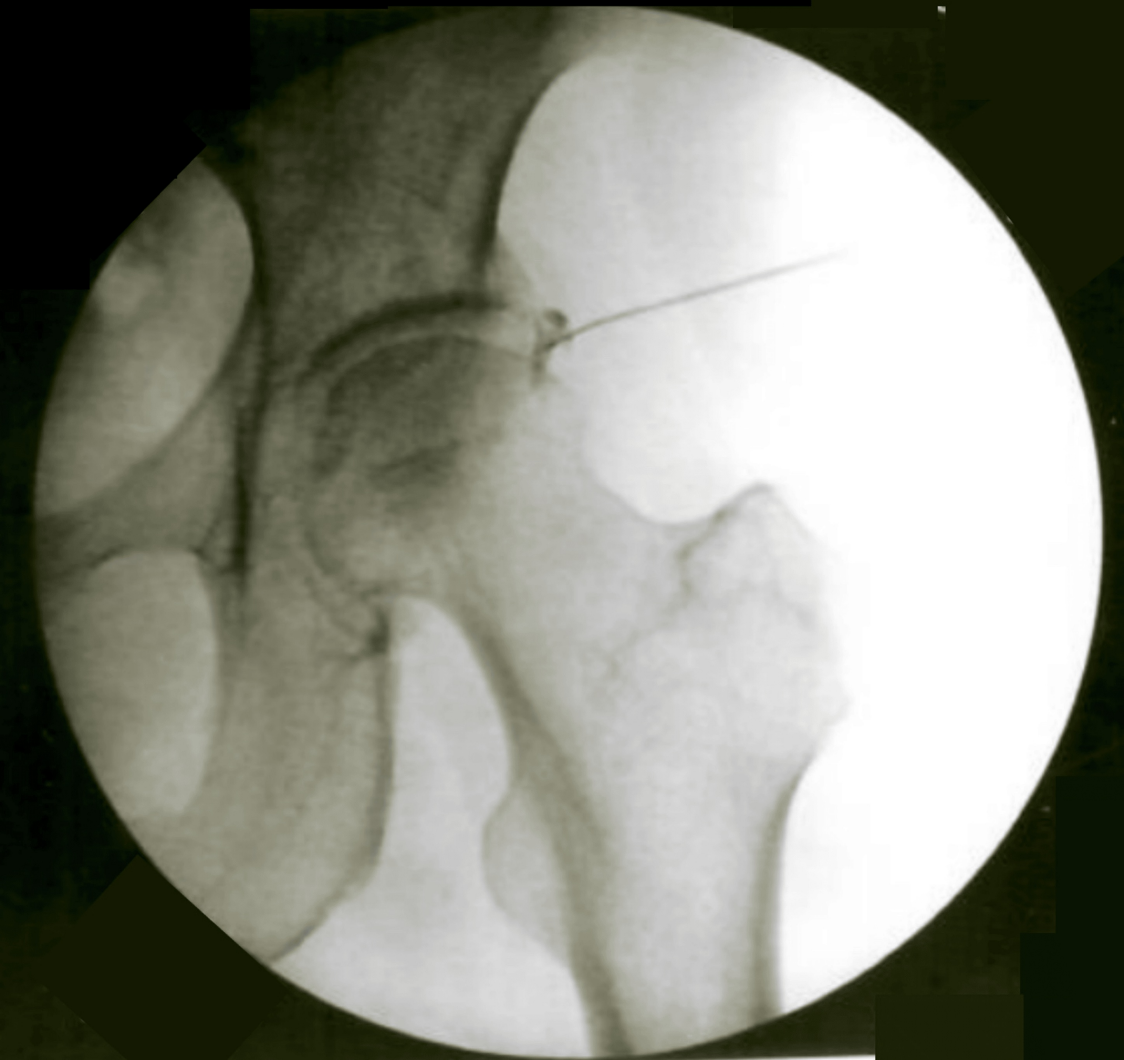
Patient 4 - left hip BMC injection Fluoroscopic image of a BMC injection in a 70-year-old female (Tönnis 1) with degenerative labral tearing and acetabular subcondrial cysts. Patient 4 reported post-procedure VAS improved from 8 to 4 with 50% SANE functional improvement. BMC, bone marrow concentrate; VAS, visual analog scale; SANE, single assessment numeric evaluation

Patient 4 was a 70-year-old female (Tönnis 1) with chronic left hip pain from a degenerative labral tear and associated acetabular subchondral cysts. MRI showed degenerative tearing of the anterior and superior labrum, cartilage injury, and acetabular subchondral cyst formation. Baseline VAS was 8. She received BMC injections (TNC=3.8×10⁸; viability=95%) to the greater trochanter, acetabulum, superior labrum, and joint capsule (Figure 4). The greater trochanter was injected with 1 cc BMC, 1 cc 10× SCP, and 1 cc 10× PLM. The acetabular IO injection included 3 cc BMC, 2 cc 10× SCP, and 2 cc 10× PLM. The superior labral region received 2 cc BMC and 1 cc 10× SCP, while the IA injection contained 1 cc BMC and 1 cc 10× PLM. At the two-month follow-up, she reported VAS improved from 8 to 4 with 50% SANE functional improvement. She continued PT and home exercises with gradual gains at four-month and six-month follow-ups, but noted residual pain with certain activities.

Case 5

**Figure 5:**
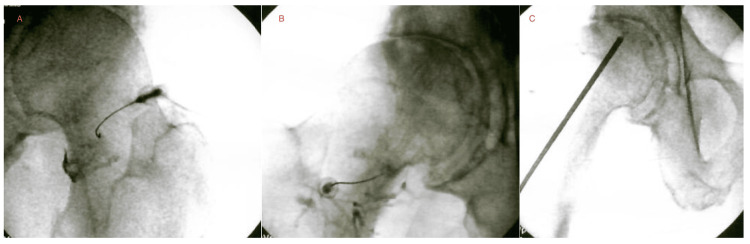
Patient 5 - bilateral hip BMC injections Fluoroscopy-guided bilateral intra-articular injections (A, B) and right hip intraosseous BMC injection (C). X-ray of the hips indicated a Tönnis score of 3. At follow-up, the patient reported VAS improvement from 9 to 2 and 70% SANE improvement in function after the second treatment. BMC, bone marrow concentrate; SANE, single assessment numeric evaluation

Patient 5 was an 81-year-old male (Tönnis 3) with chronic bilateral hip OA, moderate on the left and severe on the right, confirmed by MRI showing full-thickness cartilage loss and subchondral sclerosis. X-ray imaging showed subchondral sclerosis and narrowing of the superior and inferior aspects of the right acetabulum and narrowing of the superior aspect of the left acetabular joint. Hypertrophic changes of the femoral heads were also noted, with mild involvement on the left and moderate-to-marked involvement on the right. Baseline VAS was 9. For the right hip, BMC injections (TNC=1.4×10⁹; viability=95%) included a femoral head IO mixture of 4 cc BMC, 2 cc 10× SCP, 2 cc 10× PLM, and 2 cc 7× SCP; an acetabular IO injection of 3 cc BMC, 1 cc 10× SCP, 1 cc 10× PLM, and 5 cc 7× SCP; and an IA injection containing 1 cc BMC and 1 cc 7× SCP. The left hip received a combined superior labral and IA injection consisting of 1 cc 10× SCP, 1 cc 10× PLM, and 2 cc 7× SCP. The IO BMC injection to the right hip was followed by a second series six months later (Figure 5). After another six months, patient 5 reported VAS improved from 9 to 2 and a 70% improvement in function.

The cohort was composed of four males and one female with an average age of 67.6 and a Tönnis score of 2. All patients experienced decreased pain and overall improvement in function after IO BMC injection. Pain relief, reported as the average difference in VAS scores, was 5.2, and all patients reported at least 50% SANE functional improvement (Table 1). Adverse events were systematically reviewed at each follow-up visit and via post-procedure phone calls. No adverse effects or major complications were reported.

**Table 1:** Patient demographics and results Patients’ Tönnis scale grades, pre- and post-BMC VAS scores, and SANE functional improvement percentage. *Patient received two BMC injections six months apart. Value is after both injections. M, male; F, female; OA, osteoarthritis; BMC, bone marrow concentrate; TNC, total nucleated cell count; VAS, visual analog scale; SANE, single assessment numeric evaluation

Case Report Number	Age/Gender	Diagnosis	Tönnis Score	BMC TNC, % Viability	Follow-up intervals in months	Pre -> Post BMC VAS	Functional Improvement (SANE)
1	54 M	Left hip mild OA	1	1.5×10⁹, 87%	2, 4, 6	7 -> 3	60%
2	65 M	Right hip OA	2	2.0×10⁹, 85%	1	7 -> 2	at least 50%
3	68 M	Left hip moderate OA, elevated left pelvis	3	Information not available	1, 6	7 -> 1	60-70%
4	70 F	Torn left hip labrum, left hip OA	1	3.8×10^8^, 95%	2, 4, 6	8 -> 4	50%
5	81 M	Bilateral hip OA, moderate left, severe right	3	1.4×10⁹, 95%	3, 6, 12	9 -> 2	70%*

## Discussion

Although follow-up duration varied in our study, all five patients experienced at least 50% improvement in pain and function. This improvement was independent of age or gender, as previously reported in the literature [28,29,30]. Generally, higher OA grading is associated with a reduced response to injectables [30]. However, in our study, OA severity did not limit the benefit of BMC for pain or function. BMC is a solution containing growth factors, platelets, and biofactors. Most notably, BMC contains MSCs with the potential to differentiate into various tissues. MSCs have chondrogenic potential to stimulate cartilage growth and utilize trophic factors to suppress inflammation. Repeat MRIs post-BMC injection have demonstrated maintenance of cartilage regeneration and even increased thickness after two years [14,16,28]. In our study, repeat imaging was not performed; therefore, cartilage maintenance in our patients cannot be commented on.

Previous case series with limited sample sizes have shown symptomatic improvement in pain and function in ankle, knee, and hip OA following BMC injections [13,14,16,28-32]. A study published in *Clinical Medicine Insights* evaluated outcomes after four consecutive IA BMC hip injections over 3.5 months and reported 67% total overall improvement. Administering injections 14 days apart allows a higher dose of growth factors to enhance MSC growth and differentiation from various cell types involved in late phases of wound healing. Patients experienced incremental improvement after each treatment, with maximum benefit observed after the fourth injection [14]. Patient 5, who received two BMC injections, showed the greatest improvement in pre- and post-VAS and SANE scores compared with other patients in our study who received only one dose (Table 1).

Two years post-injection, all five patients were contacted for follow-up regarding pain and function. Three of the five patients were reached. Patient 1 reported ongoing 10% relief, with pre-BMC VAS of 7 and post-injection VAS of 5. He has not undergone other hip procedures but is exploring THA options. Patient 4 reported anterior hip pain improved by 40% and lateral pain by 10% after BMC. Two-year post-BMC VAS ranged from 6 to 7, compared with a pre-procedure VAS of 8. She had received a cortisone injection to her hip since the BMC injection, but no other procedures. Patient 5 reported no issues in his left hip but continued pain in his right hip, which further progressed at the time of injection. He was exploring right hip THA but had not received any other hip procedures since BMC as of the two-year follow-up.

This study represents an exploratory case series and should be interpreted within its inherent limitations. The small sample size (n=5) limits generalizability, and no statistical analysis or control group was included to differentiate true treatment effects from natural recovery, rehabilitation, or placebo response. Follow-up intervals were inconsistent across patients, ranging from one month to two years, and some patients underwent adjunct procedures, such as PRP and capsular distention, at different time points, which may have contributed to clinical improvement. Objective imaging or biomarker outcomes were not consistently obtained at follow-up, precluding structural assessment of joint changes. Despite these limitations, the consistent improvements in pain and function, along with the absence of adverse events across cases, highlight the potential feasibility and safety of IO BMC for hip OA. However, the lack of sustained long-term success in pain and functional outcomes underscores the need for additional research to optimize treatment protocols, including patient selection and dosing strategies. Future randomized controlled studies with standardized imaging, defined follow-up schedules, and comparison groups are needed to confirm these preliminary findings and further elucidate mechanisms of action and generalizability to the broader population in the growing field of orthobiologic and regenerative interventions for OA.

## Conclusions

This small case series suggests that IO BMC injections may represent a feasible and safe, minimally invasive option for patients with hip OA. Across five cases, consistent improvements in pain and self-reported function were observed without adverse events, providing early signals of potential clinical benefit. However, these findings should be interpreted as preliminary and exploratory given the limited sample size, variable follow-up duration, and absence of a control group. Future randomized controlled trials with standardized protocols, objective imaging, and larger patient populations are needed to confirm safety, determine efficacy, and clarify the mechanisms underlying observed improvements.
